# Identification of Suitable Reference Genes for Investigating Gene Expression in Anterior Cruciate Ligament Injury by Using Reverse Transcription-Quantitative PCR

**DOI:** 10.1371/journal.pone.0133323

**Published:** 2015-07-20

**Authors:** Mariana Ferreira Leal, Diego Costa Astur, Pedro Debieux, Gustavo Gonçalves Arliani, Carlos Eduardo Silveira Franciozi, Leonor Casilla Loyola, Carlos Vicente Andreoli, Marília Cardoso Smith, Alberto de Castro Pochini, Benno Ejnisman, Moises Cohen

**Affiliations:** 1 Departamento de Ortopedia e Traumatologia, Universidade Federal de São Paulo, 04038–032, São Paulo, SP, Brazil; 2 Disciplina de Genética, Departamento de Morfologia e Genética, Universidade Federal de São Paulo, 04023–001, São Paulo, SP, Brazil; Northwestern University, UNITED STATES

## Abstract

The anterior cruciate ligament (ACL) is one of the most frequently injured structures during high-impact sporting activities. Gene expression analysis may be a useful tool for understanding ACL tears and healing failure. Reverse transcription-quantitative polymerase chain reaction (RT-qPCR) has emerged as an effective method for such studies. However, this technique requires the use of suitable reference genes for data normalization. Here, we evaluated the suitability of six reference genes (*18S*, *ACTB*, *B2M*, *GAPDH*, *HPRT1*, and *TBP*) by using ACL samples of 39 individuals with ACL tears (20 with isolated ACL tears and 19 with ACL tear and combined meniscal injury) and of 13 controls. The stability of the candidate reference genes was determined by using the NormFinder, geNorm, BestKeeper DataAssist, and RefFinder software packages and the comparative ΔCt method. *ACTB* was the best single reference gene and *ACTB+TBP* was the best gene pair. The GenEx software showed that the accumulated standard deviation is reduced when a larger number of reference genes is used for gene expression normalization. However, the use of a single reference gene may not be suitable. To identify the optimal combination of reference genes, we evaluated the expression of *FN1* and *PLOD1*. We observed that at least 3 reference genes should be used. *ACTB+HPRT1+18S* is the best trio for the analyses involving isolated ACL tears and controls. Conversely, *ACTB+TBP+18S* is the best trio for the analyses involving (1) injured ACL tears and controls, and (2) ACL tears of patients with meniscal tears and controls. Therefore, if the gene expression study aims to compare non-injured ACL, isolated ACL tears and ACL tears from patients with meniscal tear as three independent groups *ACTB+TBP+18S+HPRT1* should be used. In conclusion, 3 or more genes should be used as reference genes for analysis of ACL samples of individuals with and without ACL tears.

## Introduction

The anterior cruciate ligament (ACL) is an important structure in the knee and is one of the most frequently injured structures during high-impact sporting activities [1,2,3]. The ACL does not heal following lesions, and surgical reconstruction is the treatment of choice in most cases [4,5]. Reconstructive surgery aims to restore the kinematics and stability of the injured knee, which allows a return to sports and may help to prevent osteoarthritis in the long term [3,5,6,7].

Some studies have been done to elucidate the molecular basis for failure of the human ACL to heal after rupture [6,8,9,10,11,12]. An improved understanding of the regulation of gene expression in normal and injured ACL will be important for guiding patient management and the development of new therapeutic options complementary to surgery.

Because of its accuracy, sensitivity, and capacity for high-throughput analysis, reverse transcription-quantitative polymerase chain reaction (RT-qPCR) is currently considered to be the gold standard technique for evaluation of gene expression [13]; furthermore, this technique is commonly used to validate data obtained by other methods [14].

A common method for obtaining reliable data through RT-qPCR is to normalize the target gene expression by using an endogenous reference gene. The use of one or more reference genes can correct biases caused by variations in the complementary DNA (cDNA) input or in the efficiency of RT or amplification. Ideally, reference genes should be stably expressed or at least vary only slightly in expression in all tissues or cells under the conditions of the experiment [15]. Normalization with unstable internal controls may result in different values and lead to erroneous results. Thus, it is necessary to meticulously evaluate the expression profiles of the candidate reference genes for each experimental system [16].

The suitability of reference genes has been evaluated in some human musculoskeletal diseases, such as shoulder instability [17], rotator cuff tears [18], osteoarthritic articular cartilage (hip and knee) [19], human lumbar vertebral endplate with Modic changes [20], and skeletal muscle with chronic degenerative changes [16]. Ayers et al. reported that the best reference genes for comparing normal and ruptured canine cranial cruciate ligament were *B2M* and *TBP* [21]. However, *18S* [8], *ACTB* [9], and *GAPDH* [10] have been used as reference genes in the study of mRNA regulation in human ACL tears.

To our knowledge, no previous studies have described the best individual or set of reference genes for gene expression analysis of samples of human ligament. In this study, we assessed the suitability of six reference genes frequently reported in the literature (*18S*, *ACTB*, *B2M*, *GAPDH*, *HPRT1*, and *TBP*) by using ACL injury samples of patients with or without concomitant meniscal tears and control samples, analyzing the gene stability with the use of five software packages and the comparative ΔCt method.

## Materials and Methods

### Patients

Tissue samples were obtained from 39 patients with ACL tears, including 20 samples from patients with isolated ACL tears and 19 samples from patients with ACL injury and concomitant meniscus injury. Arthroscopic ACL reconstruction was done on all patients. The following inclusion criteria were used: age between 18 and 50 years old, clinical and magnetic resonance imaging (MRI) diagnosis of ACL injury, and ACL lesion at the femoral insertion or disruption. The Lachman test [22], anterior drawer test [23], and pivot-shift tests [24] were used to diagnose ACL injury [7]. The McMurray [25], Apley [26], and Steinman [27] tests were used to diagnose meniscus injury [28]. Coronal and sagittal MRI views were used to identify ACL and meniscal lesions. All injuries were confirmed during the arthroscopic procedure and reclassified when necessary.

Additionally, 13 patients without any history of ACL tears were included in this study as a control group. These patients had been arthroscopically operated on for other knee injuries, such as isolated medial meniscus injury. All control patients were physically active. Table 1 displays the main clinical outcomes of the studied cases and controls.

**Table 1:** Distribution of the clinical outcomes of anterior cruciate ligament tear patients and controls.

Variable	Cases (N = 39)	Controls (N = 13)
Age at surgery, years (mean ± SD)	34 ± 11.3	38 ± 9.7
Gender (% of male)	64.1%	69.2%
Duration of condition, months (mean ± SD)	5 ± 3	8.6 ± 7.4
Mechanism (% of traumatic onset of symptoms)	94.9%	76.9%

N: number of samples; SD: standard deviation.

This study was approved by the Ethics Committee of the Universidade Federal de São Paulo, Brazil (CEP #51436). Written informed consent was obtained from all patients before specimen collection.

### Tissue samples

For the collection of tissue samples, the patients were subjected to the standard preparation for surgical ACL reconstruction. A standard arthroscopic joint evaluation was carried out, confirming the diagnosis of ACL injury or combined ACL and meniscus injury. During surgery, samples (about 5 mm^3^) of free edge from the injured ACL were collected for gene expression analysis; it is common to find remaining tissue in the ACL tear extremity [6]. The ACL tissue samples were obtained from the most proximal and anterior ACL local tear. After sample collection, the ACL reconstruction was concluded.

In the controls, similarly to the patients, a sample fragment of about 5 mm^3^ was resected from the most proximal and anterior ACL fibers in the ACL without any sign of tears.

All tissue specimens were immediately immersed in Allprotect Tissue Reagent (Qiagen, USA) and stored at -20°C until RNA extraction.

### RNA extraction

Total RNA was extracted from 10–20 mg of tissue sample using an AllPrep DNA/RNA/miRNA Mini Kit (Qiagen, USA) according to the manufacturer’s protocol. The mechanical lysis step was performed using the Tissue Lyser LT equipment (Qiagen, USA). RNA concentration and quality were immediately determined using a Nanodrop ND-1000 (Thermo Scientifc, USA) and the integrity of the RNA was verified by gel electrophoresis on a 1% agarose gel. Aliquots of the total RNA were stored at -80°C until further use.

### RT-qPCR

RT-qPCR gene expression quantifications were performed according to MIQE guidelines [29]. Only RNA samples with the optical density (OD)_260/280_ > 1.8 were used, following the MIQE protocol.

First, cDNA was synthesized from 200 ng of RNA using a High-Capacity cDNA Reverse Transcription Kit (Life Technologies, USA) according to the manufacturer’s protocol.

To detect the range of expression of the six candidate reference genes, reactions were performed with 75 ng of cDNA input using TaqMan Low-Density Array (TLDA) cards (Life Technologies, USA) and ViiA 7 Real-Time PCR System (Life Technologies, USA). Only inventoried TaqMan Gene Expression Assays (Life Technologies, USA) were chosen for gene expression analysis. The final volume in each TLDA well is approximately 1 μl. All reactions were performed in triplicate.

To identify the best combination of reference genes, we also quantified the mRNA expression of targets genes, *FN1* and *PLOD1*, using the candidate reference genes for normalization. Fibronectin (FN), a large multidomain glycoprotein found in all vertebrates, plays a vital role in cell adhesion, tissue development, and wound healing [30]. The lysyl hydroxylases 1 (encoded by *PLOD1*) promote extracellular matrix (ECM) structural stability and maturation by promoting inter- and intramolecular cross-links and the addition of carbohydrate moieties to ECM molecules.[31,32]. Therefore, *FN1* and *PLOD1* may have a role in ACL tears and healing.

For each sample, the candidate reference and target genes were assayed on the same card to exclude technical variations. The 6 reference genes and target genes are summarized in Table 2.

**Table 2:** Summary of six reference genes and target genes.

Gene symbol	Name	Gene function	Assay*
*18S*	Eukaryotic 18S rRNA	Ribosome subunit	Hs99999901_s1
*ACTB*	Beta-actin	Cytoskeletal structural protein	Hs01060665_g1
*B2M*	Beta-2-microglobulin	Beta-chain of major histocompatibility complex class I molecules	Hs00984230_m1
*GAPDH*	Glyceraldehyde-3-phosphate dehydrogenase	Oxidoreductase in glycolysis and gluconeogenesis	Hs02758991_g1
*HPRT1*	Hypoxanthine phosphoribosyl-transferase	Purine synthesis in salvage pathway	Hs02800695_m1
*TBP*	TATA box binding protein	RNA polymerase II, transcription factor	Hs00427620_m1
*FN1*	Fibronectin 1	Extracellular matrix structural protein	Hs00365052_m1
*PLOD1*	Lysyl hydroxylases 1	Collagen cross-linking	Hs00609368_m1

*TaqMan probes were purchased as assays-on-demand products for gene expression (Life Technologies, USA).

The relative threshold method (Crt method) was applied, which is a robust method that sets a threshold for each curve individually based on the shape of the amplification curve, regardless of the height or variability of the curve during its early baseline fluorescence. The expression of *FN1* gene across the samples was calculated using the equation ΔCrt, in which [ΔCrt = tsarget gene (*FN1* or *PLOD1*) Crt—the mean of reference genes Crt]. A lower cycle threshold value (Crt) indicates higher gene expression.

### Analysis of reference gene expression stability

We categorized the tissue samples into the following 7 groups: 1) isolated ACL tear samples (N = 20); 2) ACL tear samples of patients with a concomitant meniscal tear (N = 19); 3) ACL control samples (N = 13); 4) all injured ACL (N = 39); 5) isolated ACL tear samples and controls (N = 33); 6) ACL tear samples of patients with a concomitant meniscal tear and controls (N = 32); 7) all ACL samples (N = 52). Typically, gene expression studies compare transcript levels between case (i.e., the injured tissue) and control samples [9], therefore we created the groups #5, #6 and #7. However, some researchers have been investigated a possible association between gene expression and clinical variables [8,10], therefore we created the groups #1, #2 and #4. In addition, the group composed by only controls (group #3) was created since the understanding of gene expression regulation in non-injured ligaments is still necessary.

For comparisons of candidate reference gene stability we used the software programs NormFinder (http://www.mdl.dk/publicationsnormfinder.htm), geNorm (http://medgen.ugent.be/~jvdesomp/genorm/ http://medgen.ugent.be/~jvdesomp/genorm/), BestKeeper1 (http://www.gene-quantifcation.de/bestkeeper.html) and DataAssist (http://www.lifetechnologies.com/us/en/home/technical-resources/software-downloads/dataassist-software.html) and the comparative ΔCt method [33]. We also used the RefFinder software (http://www.leonxie.com/referencegene.php) which integrates the results of geNorm, Normfinder, BestKeeper, and the comparative ΔCt method to compare and rank the tested candidate reference genes.

NormFinder accounts for both intra- and inter-group variations when evaluating the stability of each single reference gene [34]. The stability values and standard errors are calculated according to the transcription variation of the reference genes. Stably expressed genes, which have low variation in expression levels, present low stability values. NormFinder analysis also calculated the stability value for two reference genes.

geNorm calculates the expression stability value (M) for each gene based on the average pairwise expression ratio between a particular gene and all other reference genes. geNorm sequentially eliminates the gene that shows the highest variation relative to all the other genes based on paired expression values in all the studied samples. The most stably expressed gene yields the lowest M value, and then the two most stable reference genes are determined by stepwise exclusion of the least stable gene [35]. Because of the elimination process, geNorm cannot identify a single suitable reference gene, and ends up by suggesting a pair of genes that shows high correlation and should be suitable for normalization of qPCR studies.

Bestkeeper was used to rank the 6 reference genes based on the standard deviation (SD) and coefficient of variance (CV) expressed as a percentage of the cycle threshold (Ct) level [36]. The more stable reference gene presents the lowest CV and SD. Bestkeeper also uses a statistical algorithm wherein the Pearson correlation coefficient for each candidate reference gene pair is calculated along with the probability of correlation significance of the pair.

DataAssist software provided a metric to measure reference gene stability based on the geNorm algorithm. In contrast to the other programs, DataAssist uses RQ to calculate the stability value of individual candidate reference genes. The lower score represents the more stable the control.

The comparative ΔCt method is based on the comparing relative expression of pairs of possible reference genes within each sample. The stability of the candidate housekeeping genes is ranked according to reproducibility of the gene expression differences among studied samples.

Lastly, RefFinder assigns an appropriate weight to an individual gene and calculated the geometric mean of their weights for the overall final ranking based on the rankings from geNorm, Normfinder, BestKeeper, and the comparative ΔCt.

GenEx software (http://genex.gene-quantifcation.info/) was used to determine the optimal number of reference genes by calculating the accumulated standard deviation (Acc.SD). If larger number of reference genes is used, random variation among the genes’ expression partially cancel reducing the SD. A minimum in the Acc.SD plot indicate the number of reference genes that give the lowest SD.

### Statistical analysis

To compare *FN1* and *PLOD1* expression between the groups, we first verified the distribution of the data using the Kolmogorov-Smirnov normality test for the determination of the appropriate tests for the subsequent statistical comparisons. *FN1* and *PLOD1* expression (ΔCrt) was normally distributed. Therefore, the independent T-test was performed to compare *FN1* and *PLOD1* expression between the studied groups, and the values are shown as the mean ± standard deviation (SD). A p-value of < 0.05 was considered statistically significant.

## Results

### Reference gene expression levels

**Fig 1:**
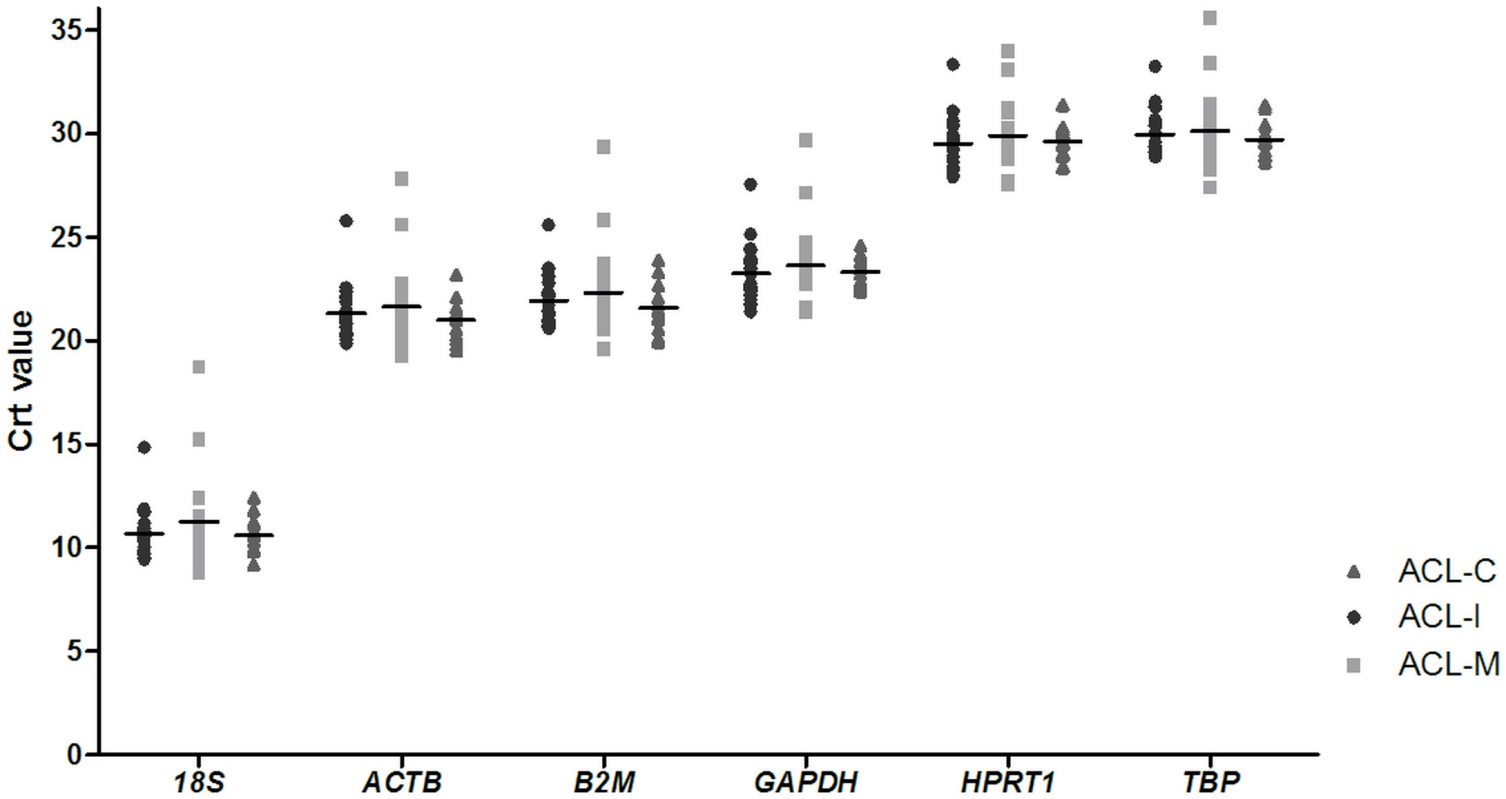
RT-qPCR detection of the expression levels of six reference genes. A lower cycle threshold value (Crt) indicates higher gene expression. ACL-I: isolated anterior cruciate ligament tear samples; ACL-M: anterior cruciate ligament tear samples of patients with concomitant meniscal tear; ACL-C: anterior cruciate ligament samples of controls.

Fig 1 presents the distribution of Crt values for each of the 6 candidate reference genes. These genes showed a wide range of expression levels, with *18S* presenting the highest expression level (mean Crt value ± SD: 10.85 ± 1.63). In contrast, *TPB* (29.96 ± 1.36) and *HPRT1* (29.69 ± 1.30) had the lowest expression levels in the ACL samples.

### Reference gene expression stability

S1 Table shows the stability value ranking of the single candidate reference genes, as determined by the different software packages and the comparative ΔCt method. In our analysis, all the reference genes for all the groups presented M values less than the geNorm threshold of 1.5, which is considered as stable (S1 Table). However, *B2M* presented a high SD of Crt (SD = 1.12) in the analysis of all samples with the use of the BestKeeper software, in which any studied gene with SD higher than 1 can be considered inconsistent.

Although neither the software packages nor the comparative ΔCt method suggested the same rank of reference genes in the studied sample groups, the methods applied did generate similar rankings of reference gene stability for each analysis group (S1 Table).

Table 3 shows the most suitable reference gene based on the different software packages. In the present study, *ACTB* was found to be the most suitable reference gene for the study of ACL samples. As previously described, gene expression studies typically compare transcript levels between injured and non-injured tissue samples. When the isolated ACL tear samples and the controls were evaluated together, *ACTB*, followed by *18S*, was found to be the most suitable reference gene. When the ACL tear samples of patients with a concomitant meniscal tear and the controls were considered, *ACTB*, followed by *TBP*, was the most stable gene. When all ACL tear samples and all control samples were considered, *ACTB*, followed by *TBP*, was also the most stable gene (Table 3, S1 Table).

**Table 3:** Best reference gene for each group of sample.

Groups	Best reference gene by each method					
	NormFinder^a^	GeNorm	BestKeeper	DataAssist	ΔCt method	RefFinder
Isolated ACL tear samples	***HPRT1***	***ACTB*** */18S*	*TPB*	***ACTB***	***HPRT1***	***HPRT1***
ACL tear samples of patients with a concomitant meniscal tear	***ACTB***	***ACTB*** */TBP*	*HPRT1*	***ACTB***	***ACTB***	***ACTB***
ACL controls	***ACTB***	*HPRT1/GAPDH*	*TPB*	***ACTB***	***ACTB***	***ACTB***
All injured ACL samples	***ACTB***	***ACTB*** */TBP*	*HPRT1*	***ACTB***	***ACTB***	***ACTB***
Isolated ACL tear samples and controls	***18S*** *	***ACTB*** */* ***18S***	*TPB*	***ACTB***	***ACTB***	***ACTB***
ACL tear samples of patients with a concomitant meniscal tear and controls	***ACTB***	***ACTB*** */18S*	*HPRT1*	***ACTB***	***ACTB***	***ACTB***
All ACL samples	***ACTB***	***ACTB*** */18S*	*HPRT1*	***ACTB***	***ACTB***	***ACTB***

^a^Best reference gene determined considering the intragroup and intergroup variation.

*When the intragroup and intergroup variation was not considered, *ACTB* was the best reference gene by NormFinder. Bold letters: best pairs of reference genes by more than one of the methods commonly used (Normfinder, GeNorm, BestKeeper, DataAssist, ΔCt method and RefFinder). ACL: anterior cruciate ligament.

When each group of ACL samples was evaluated individually, *HPRT1*, followed by *ACTB*, was observed to be the most stable gene for the isolated ACL tear samples. *ACTB* was also identified as the most stable gene in the ACL tear samples of patients with a concomitant meniscal tear and in the control samples. When all injured ACL samples were considered, *ACTB* was also identified as the most stable gene (Table 3, S1 Table).

### Analysis of the best combinations of reference genes

Table 4 shows the best combinations of reference genes, as suggested by the software packages, the comparative ΔCt method, and visual inspection of all the ranks generated by these analyses. Overall, the *ACTB* + *TBP* and *ACTB + 18S* pairs of genes were the most frequently identified. *ACTB + 18S* was the most frequently identified pair in the analysis of samples of (1) isolated ACL tear samples and (2) isolated ACL tear samples and controls. In contrast, *ACTB + TBP* was the most frequently identified pair in the analysis of samples of (1) ACL tear samples of patients with a concomitant meniscal tear, (2) all injured ACL samples, (3) ACL tear samples of patients with a concomitant meniscal tear and controls, and (4) all ACL samples. In addition, *GAPDH* + *HPRT1* was the most frequently identified pair of reference genes in the analysis of control samples.

**Table 4:** Best combination of reference genes for each group of sample.

Groups	Best pair of reference genes by software				Top genes by ΔCt method	Top genes by RefFinder	Best pair of reference genes^c^	Best trio of reference genes^c^
	NormFinder^a^	GeNorm	BestKeeper^b^	DataAssist				
Isolated ACL tear samples	***ACTB+HPRT1***	***ACTB+18S***	***ACTB+18S***	***ACTB+18S***	***ACTB+HPRT1***	*HPRT1+18S*	*ACTB+HPRT1*	*ACTB+HPRT1+18S*
ACL tear samples of patients with a concomitant meniscal tear	***ACTB+TBP***	***ACTB+TBP***	***ACTB+TBP***	***ACTB+TBP***	***ACTB+TBP***	***ACTB+TBP***	*ACTB+TBP*	*ACTB+TBP+18S*
ACL controls	*ACTB+18S*	***GAPDH+HPRT1***	***GAPDH+HPRT1***	***GAPDH+HPRT1***	*ACTB+HPRT1*	*ACTB+TBP*	*ACTB+HPRT1*	*ACTB+HPRT1+TBP*
All injured ACL samples	***ACTB+18S*** *	***ACTB+TBP***	***ACTB+TBP***	***ACTB+18S***	***ACTB+TBP***	***ACTB+TBP***	*ACTB+TBP*	*ACTB+TBP+18S*
Isolated ACL tear samples and controls	***ACTB+HPRT1*** **	***ACTB+18S***	***ACTB+18S***	***ACTB+18S***	***ACTB+HPRT1***	***ACTB+18S***	*ACTB+18S*	*ACTB+HPRT1+18S*
ACL tear samples of patients with a concomitant meniscal tear and controls	***ACTB*+*TBP***	***ACTB+18S***	***ACTB+18S***	***ACTB+18S***	***ACTB+TBP***	***ACTB+TBP***	***ACTB+TBP***	*ACTB+TBP+18S*
All ACL samples	***ACTB*+*TBP***	***ACTB+18S***	***ACTB+18S***	***ACTB+18S***	***ACTB+TBP***	***ACTB+TBP***	***ACTB+TBP***	*ACTB+TBP+18S*

^a^Best combination of two genes determined considering the intragroup and intergroup variation;

^b^Best combination of two genes determined considering the correlation values (r);

^c^Best combination is based in a visual inspection of all the ranks generated by the four software.

*When the intragroup and intergroup variation was not considered, *ACTB+TBP* was the best pair of reference gene by NormFinder.

**When the intragroup and intergroup variation was not considered, *ACTB+18S* was the best pair of reference gene by NormFinder. Bold letters: best pairs of reference genes by more than one of the methods commonly used (Normfinder, GeNorm, BestKeeper, DataAssist, ΔCt method and RefFinder). Underlined letters: best pairs of reference genes by visual inspection and of the methods commonly used. ACL: anterior cruciate ligament.

The NormFinder, geNorm, DataAssist, and BestKeeper software packages indicated only up to 2 genes as the best combination of reference genes. Visual inspection of all the ranks generated by the software and comparative ΔCt method indicated that *ACTB + TBP* + *18S*, followed by *ACTB + HPRT1 + 18S*, was the best trio of reference genes.

**Fig 2:**
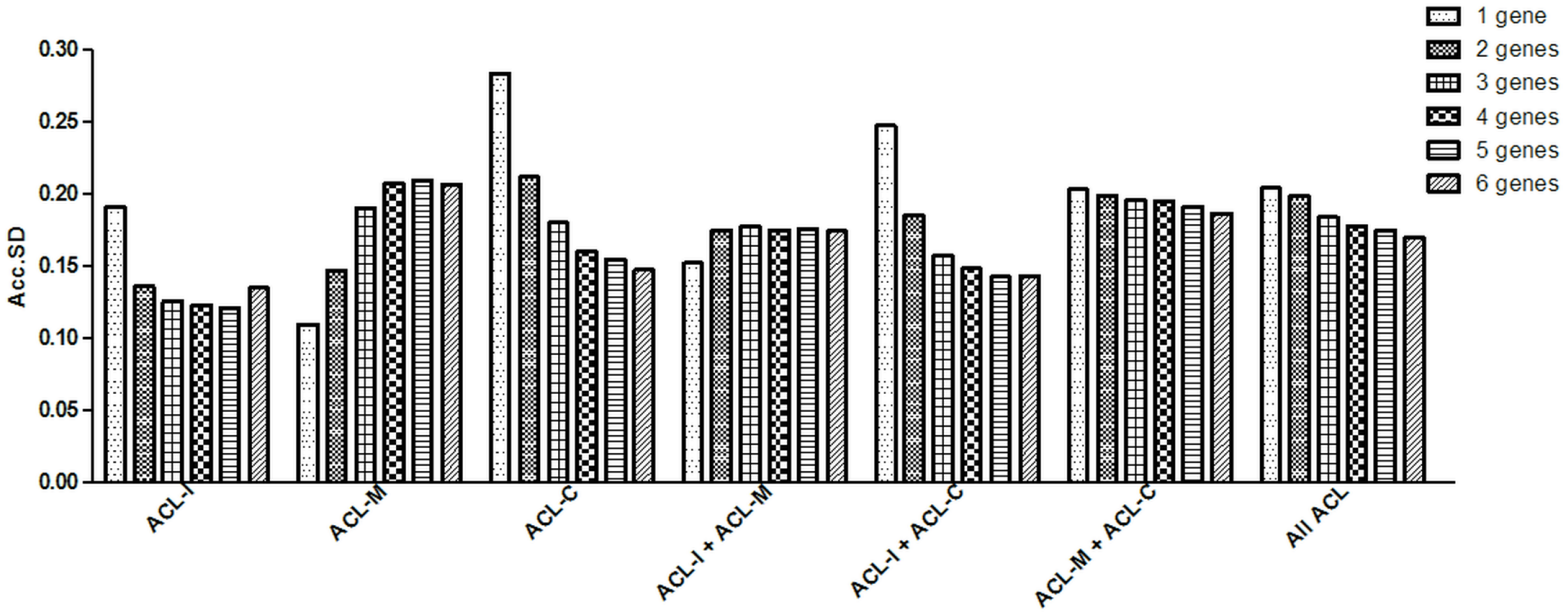
Accumulated standard deviation for the 6 reference genes in ACL samples. Lower values of accumulated standard deviation (Acc.SD) indicate the optimal number of reference gene as estimated by the GenEx software package. ACL-I: isolated anterior cruciate ligament tear samples; ACL-M: anterior cruciate ligament tear samples of patients with concomitant meniscal tear; ACL-C: anterior cruciate ligament samples of controls.

We used the GenEx software package to determine whether reliable normalization would require more than 2 reference genes. In this analysis, the optimal number of reference genes was indicated by the lowest SD. In all analyses, the Acc.SD of 2 reference genes did not differ by more than 0.1 from the observed metric when using more than 2 genes (Fig 2). However, in the analysis of ACL control samples, the Acc.SD of 1 reference gene was more than 0.1 from the observed metric when using more than 3, 4, 5, or 6 genes (Fig 2). Moreover, in the analysis of isolated ACL tear samples and controls, the Acc.SD of 1 reference gene was more than 0.1 from the observed metric when using 5 or 6 genes (Fig 2). Conversely, in the analysis of ACL tear samples of patients with a meniscal tear, the lowest Acc.SD was observed when only one reference gene (*ACTB*) was used. In this group of samples, we observed that the Acc.SD of 6 reference genes was more than 0.1 from the metric observed with 1 gene (Fig 2).

### Effects of reference gene choice

To evaluate the effect of appropriate reference gene selection, an expression analysis was done by comparing the data from (1) ACL tear samples of patients with and without a concomitant meniscal tear, (2) isolated ACL tear samples and controls, (3) ACL tear samples of patients with a concomitant meniscal tear and controls, and (4) injured ACL samples and controls. This analysis was done with *FN1* and *PLOD1* as the target gene. The above-mentioned most frequently identified pairs (*ACTB + TBP* and *ACTB+18S*) were used as reference genes. Gene expression analysis was also done by using 3 reference genes (*ACTB + TBP* + *18S* and *ACTB + HPRT1 + 18S*), 4 reference genes *(ACTB + TBP* + *18S* + *HPRT1*), and only *18S* [8], *ACTB* [9], or *GAPDH* [10], as previously described in the literature.

**Fig 3:**
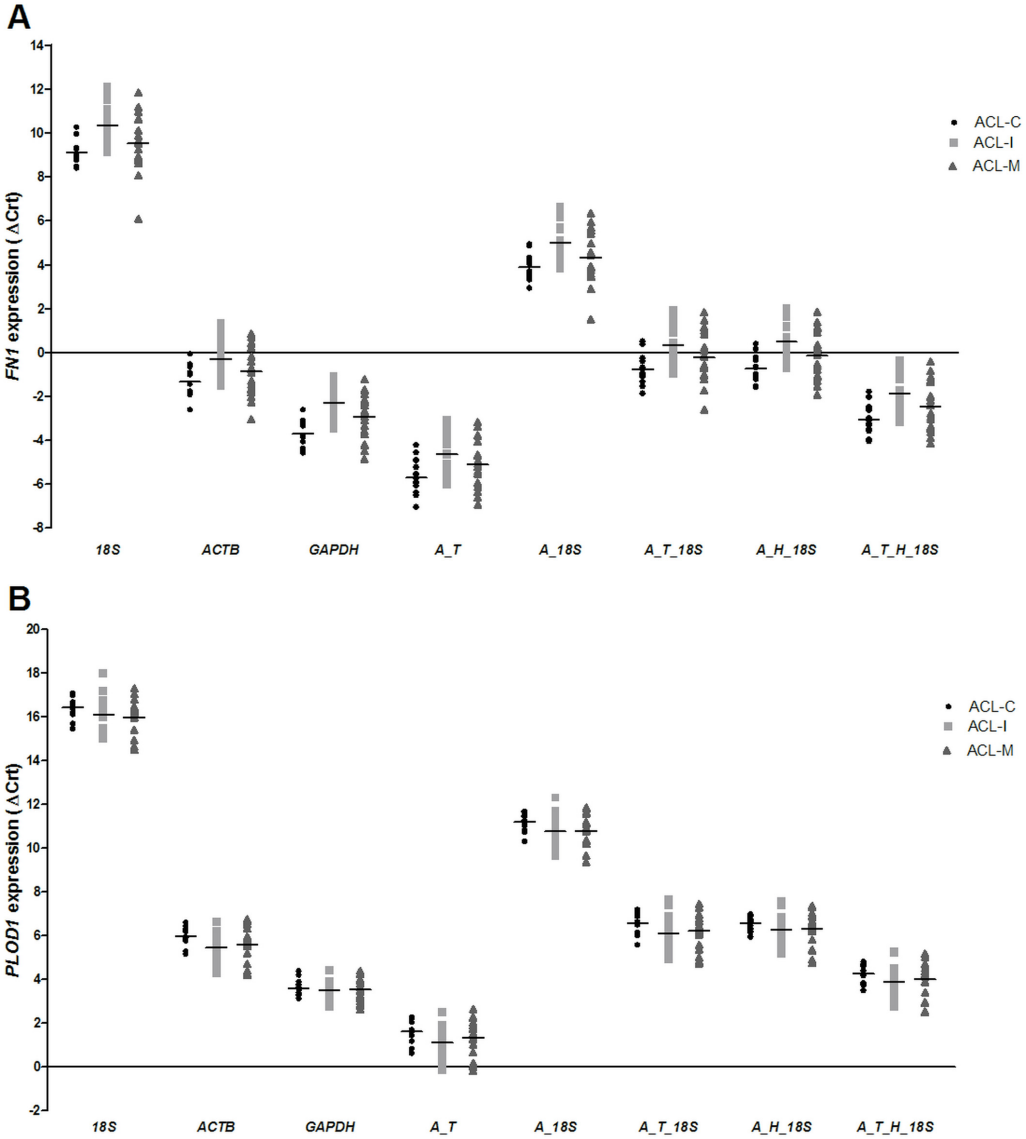
*FN1* (A) and *PLOD1* (B) expression normalized by different combinations of candidate reference genes in anterior cruciate ligament specimens. *18S*: target expression normalized by *18S*; *ACTB*: target expression normalized by *ACTB*; *GAPDH*: target expression normalized by *GAPDH; A_T*: target expression normalized by *ACTB + TBP*; *A_18S*: target expression normalized by *ACTB + 18S*; *A_T_18S*: *FN1* expression normalized by *ACTB + TBP + 18S*; *A_H_18S*: target expression normalized by *ACTB + HPRT1 + 18S*; *A_T_H_18S*: target expression normalized by *ACTB + TBP + HPRT1 + 18S*; ACL-I: isolated anterior cruciate ligament tear samples; ACL-M: anterior cruciate ligament tear samples of patients with concomitant meniscal tear; ACL-C: anterior cruciate ligament samples of controls.

Although the normalized expression quantities differed between the various combinations of reference genes, the distributions of the targets gene expression in the studied samples were similar (Fig 3).

Table 5 shows the *FN1* expression when the different reference gene combinations were used for data normalization. The *FN1* expression was significantly reduced in the ACL tear samples compared with the controls (p < 0.05), as well as in the isolated ACL tear samples compared with the controls (p < 0.05).

**Table 5:** *FN1* expression normalized by different combinations of reference genes in the anterior cruciate ligament samples.

Reference genes	*FN1* expression (ΔCrt; mean ± SD)^a^											
	ACL-I	ACL-M	p-value	ACL-I	ACL-C	p-value	ACL-M	ACL-C	p-value	ACL-I + ACL-M	ACL-C	p-value
*18S*	14.46 ± 1.38	13.88 ± 1.15	0.030*	14.46 ± 1.38	12.96 ± 1.71	<0.001*	13.88 ± 1.15	12.96 ± 1.71	0.223	14.17 ± 1.29	12.96 ± 1.71	0.001*
*ACTB*	3.81 ± 1.40	3.41 ± 1.35	0.094	3.81 ± 1.39	2.53 ± 1.82	0.001*	3.51 ± 1.35	2.53 ± 1.82	0.181	3.66 ± 1.37	2.53 ± 1.82	0.017*
*GAPDH*	1.83 ± 1.42	1.45 ± 1.21	0.029*	1.84 ± 1.42	0.15 ± 1.61	<0.001*	1.45 ± 1.21	0.15 ± 1.61	0.021*	1.65 ± 1.32	0.15 ± 1.61	<0.001*
*ACTB + TBP*	-0.52 ± 1.42	-0.75 ± 1.38	0.194	-0.52 ± 1.42	-1.83 ± 1.75	0.002*	-0.75 ± 1.38	-1.83 ± 1.75	0.117	-0.63 ± 1.38	-1.83 ± 1.75	0.011*
*ACTB + 18S*	9.13 ± 1.38	8.69 ± 1.23	0.049*	9.13 ± 1.38	7.75 ± 1.76	<0.001*	8.69 ± 1.23	7.75 ± 1.76	0.220	8.92 ± 1.31	7.75 ± 1.76	0.016*
*ACTB + TBP + 18S*	4.47 ± 1.40	4.13 ± 1.28	0.082	4.47 ± 1.40	3.10 ± 1.72	0.001*	4.13 ± 1.28	3.10 ± 1.73	0.153	4.31 ± 1.34	3.10 ± 1.72	0.011*
*ACTB + HPRT1 + 18S*	4.63 ± 1.39	4.22 ± 1.35	0.045*	4.62 ± 1.39	3.13 ± 1.74	<0.001*	4.21 ± 1.35	3.13 ± 1.73	0.066	4.42 ± 1.37	3.13 ± 1.74	0.004*
*ACTB + TBP + HPRT1 + 18S*	2.56 ± 1.40	1.91 ± 1.37	0.071	2.26 ± 1.40	1.91 ± 1.37	<0.001*	1.911 ± 1.37	0.81 ± 1.72	0.089	2.09 ± 1.38	0.81 ± 1.72	0.005*

^a^A lower cycle threshold value (Crt) indicates higher gene expression.

*p < 0.05 by independent T-test.

SD: standard deviation; ACL-I: isolated anterior cruciate ligament samples; ACL-M: anterior cruciate ligament samples of patients with concomitant meniscal tear; ACL-C: anterior cruciate ligament samples of controls.

On the other hand, the *FN1* expression was significantly reduced in the ACL tear samples of patients with a concomitant meniscal tear compared with the controls only when using *GAPDH* (p = 0.021) as reference gene.

When the isolated ACL tear samples were compared with the ACL tear samples of patients with meniscal tears, *FN1* was observed to be significantly different between the groups when its expression was normalized by *18S* (p = 0.030), *GAPDH* (p = 0.029), *ACTB + 18S* (p = 0.049), and *ACTB + HPRT1 + 18S* (p = 0.045).

Table 6 shows the *PLOD1* expression when the different reference gene combinations were used for data normalization. The *PLOD1* expression was significantly increased in the ACL tear samples compared with the controls only when *ACTB* (p = 0.008), *ACTB + 18S* (p = 0.013) and *ACTB + HPRT1 + 18* (p = 0.049) were used for its expression normalization. Moreover. *PLOD1* was observed to be significantly different between the isolated ACL tear samples and controls when its expression was normalized by *ACTB* (p = 0.009) and *ACTB + 18S* (p = 0.038).

**Table 6:** *PLOD1* expression normalized by different combinations of reference genes in the anterior cruciate ligament samples.

Reference genes	*PLOD1* expression (ΔCrt; mean ± SD)^a^											
	ACL-I	ACL-M	p-value	ACL-I	ACL-C	p-value	ACL-M	ACL-C	p-value	ACL-I + ACL-M	ACL-C	p-value
*18S*	16.09 ± 0.79	15.97 ± 0.82	0.628	16.09 ± 0.79	16.41 ± 0.46	0.202	15.97 ± 0.82	16.41 ± 0.46	0.089	16.03 ± 0.79	16.41 ± 0.46	0.113
*ACTB*	5.45 ± 0.67	5.59 ± 0.76	0.523	5.45 ± 0.67	5.98 ± 0.43	0.009*	5.60 ± 0.76	5.98 ± 0.43	0.113	5.52 ± 0.71	5.98 ± 0.43	0.008*
*GAPDH*	3.48 ± 0.40	3.54 ± 0.54	0.718	3.48 ± 0.40	3.60 ± 0.39	0.399	3.54 ± 0.54	3.60 ± 0.40	0.705	3.51 ± 0.47	3.60 ± 0.39	0.515
*ACTB + TBP*	1.12 ± 0.80	1.34 ± 0.84	0.399	1.12 ± 0.80	1.62 ± 0.54	0.056	1.34 ± 0.84	1.62 ± 0.54	0.299	1.23± 0.82	1.62 ± 0.54	0.111
*ACTB + 18S*	10.77 ± 0.72	10.78 ± 0.75	0.962	10.77 ± 0.72	11.19 ± 0.40	0.038*	10.78 ± 0.75	11.20 ± 0.40	0.082	10.78 ± 0.72	11.19 ± 0.40	0.013*
*ACTB + TBP + 18S*	6.11 ± 0.78	6.21 ± 0.79	0.674	6.11 ± 0.78	6.55 ± 0.48	0.055	6.22 ± 0.79	6.55 ± 0.48	0.186	6.16 ± 0.78	6.55 ±0.48	0.098
*ACTB + HPRT1 + 18S*	6.26 ± 0.69	6.30 ± 0.76	0.857	6.26 ± 0.69	6.59 ± 0.34	0.085	6.31 ± 0.76	6.59 ± 0.34	0.169	6.28 ± 0.71	6.58 ± 0.34	0.049*
*ACTB + TBP + HPRT1 + 18S*	3.89 ± 0.75	4.00 ±0.80	0.671	3.89 ± 0.75	4.26 ± 0.43	0.089	4.00 ± 0.80	4.26 ± 0.43	0.305	3.95 ± 0.77	4.25 ± 0.43	0.079

^a^A lower cycle threshold value (Crt) indicates higher gene expression.

*p < 0.05 by independent T-test. SD: standard deviation; ACL-I: isolated anterior cruciate ligament samples; ACL-M: anterior cruciate ligament samples of patients with concomitant meniscal tear; ACL-C: anterior cruciate ligament samples of controls.

## Discussion

RT-qPCR is one of the most commonly used approaches in functional genomics research, and its use in gene expression analysis may become routine. To minimize the influence of differences in mRNA extraction, RT, and PCR [37] between samples, it is necessary to normalize the target gene expression by a known factor. Consequently, the use of suitable reference genes with stable expression in the studied tissue (normal and/or injured) is essential for effective data normalization and the acquisition of accurate and meaningful biological data.

Reference genes have been described for RT-qPCR studies on several diseases and tissues [16,19,20,38,39,40,41,42]. Recently, our group identified the most stable reference genes in the glenohumeral capsule of patients with and without shoulder instability [17] and in patients with and without rotator cuff tears [18]. To the best of our knowledge, no previous study has aimed to identify suitable reference genes for gene expression analyses by quantitative approaches in human ACL.

In the present study, we used 5 software packages (NormFinder, geNorm, BestKeeper, DataAssist, and RefFinder) and the comparative ΔCt method to evaluate the stability of reference gene expression. Because each analysis uses distinct algorithms, different results can be expected. Therefore, it is important to use more than one software package or method to identify the most suitable reference genes among a set of candidates. Although the analyses differed in their rankings of reference gene stability and in their identification of the most suitable gene pair, at least two programs produced results that showed agreement among almost all the analyses. Our results indicate that the use of 5 statistical tools and the comparative ΔCt method aids in the identification of the best reference genes.

Surprisingly, Normfinder, geNorm and BestKeeper from RefFinder did not yield the same outcome obtained from the NormFinder, geNorm, and Bestkeeper interface (data not shown), probably due to the different versions of the algorithm. This lack of agreement was previously reported in the literature [43].

All the reference genes in this study presented an M value less than the geNorm threshold of 1.5, which is considered as stable under the different experimental conditions tested. However, *B2M* presented a high SD of Crt in the analysis of all samples with the use of the BestKeeper software. Therefore, these reference genes should not be used in an analysis involving different conditions of ACL (injured and non-injured).

Overall, *ACTB* seemed to be the most suitable gene based on the analyses of different groups. This reference gene was used in a previous study on injured and non-injured human ACL samples [9]. Although an earlier study on ACL tear samples used *GAPDH* as a reference gene [10], our analysis revealed that this gene is not the most stable in this type of tissue sample. *18S* was also previously used as reference gene in gene expression studies on human ACL tears [8]; however, this gene was observed to be the most stable only in some analyses with the geNorm software and in the analysis of isolated ACL tear samples and controls with the use of NormFinder.

It is increasingly clear that in most situations, a single reference gene is not sufficiently stable [44]. Here, we observed that the Acc.SD of 1 reference gene was more than 0.1 from the observed metric when using more than 3 or more genes in the analysis of ACL control samples and when using 5 or 6 genes in the analysis of isolated ACL tear samples and controls. The reproducibility of real-time PCR equipment is rarely less than 0.1 cycle (estimated as the SD of technical replicates); meanwhile, our results reinforce that the use of a single reference gene may not be suitable, at least when a control group of non-injured ACL samples is investigated.

Although different combinations of reference genes were determined as the most suitable for the various analysis groups, *ACTB* + *TBP* and *ACTB + 18S* were the most frequently identified pairs, and *ACTB* + *HPRT1* + *18S* and *ACTB + TBP* + *18S* were the most frequently identified trios. The selection of the appropriate combination of reference genes should consider the group of ACL samples that will be investigated.

To identify the best combination of reference genes, we evaluated the *FN1* and *PLOD1* expression in samples of ACL tissue from the cases and controls. The statistical comparison revealed that the *FN1* expression differed between the isolated ACL tear samples and the controls, as well as when all the ACL tear samples were compared with the controls. When the ACL tear samples of patients with meniscal tears were compared with the controls, no significant difference was observed, except when the *FN1* expression was normalized only by *GAPDH*, which is not the most stable gene in the ACL samples. Therefore, our results reinforce that *GAPDH* is not the most suitable reference gene for gene expression studies on ACL tears.

In the present study, the *FN1* expression was significantly different between isolated ACL tear samples and the ACL samples of patients with meniscal tears when *18S*, *GAPDH*, *ACTB + 18S*, or *ACTB + HPRT1 + 18S* were used for expression normalization. Although the mean *FN1* expression was slightly different between the isolated ACL tear samples and the ACL samples of patients with meniscal tears, we did not have the statistical power to prove this difference in the studied sample because no significant difference was observed between these two groups of samples when the best pair (*ACTB + TBP*), the best trio (*ACTB + TBP + 18S*), or four reference genes were used. The patterns of meniscal injury after impact trauma resulting in ACL rupture are not well understood. An unconstrained high-intensity impact on the tibiofemoral joint can lead to meniscal damage in conjunction with ACL ruptures [45]. This fact seems to contribute to the larger heterogeneity observed in the ACL samples of patients with meniscal tears even when more suitable reference genes were used for *FN1* normalization. Moreover, this larger heterogeneity may also explain why the *FN1* expression did not differ between the ACL samples of patients with meniscal tears and the controls.

Concerning *PLOD1* expression, it was significantly different between ACL tear samples compared with the controls only when *ACTB*, *ACTB + 18S* and *ACTB + HPRT1 + 18* were used as reference genes. Moreover. *PLOD1* was observed to be significantly different between the isolated ACL tear samples and controls when its expression was normalized by *ACTB* and *ACTB + 18S*. In these set of analyses, we did not observed a significant difference between the groups when the best trio (*ACTB + TPB + 18* and *ACTB + HPRT1 + 18*, respectively) or four reference genes were used. Therefore, the use of one, two, three, or more reference genes may lead to differences in the statistical analysis result of some group comparisons. *PLOD1* expression analysis reinforce that the selection of the appropriate normalization should consider the group of ACL samples that will be investigated.

Furthermore, our results also show that the use of only two reference genes may be not suitable for some ACL gene expression studies. *ACTB + 18S* was the best pair for the analysis involving isolated ACL tear samples and controls according most of the software, with the exception of NormFinder and the classic ΔCt method. NormFinder is the only software that takes in account the intergroup variation. When the intergroup variation was not considered, *ACTB + 18S* was also the best pair of reference gene by NormFinder in this group of samples. Thus, although *ACTB* and *18S* seem to be stable, their expression may present some variation between isolated ACL tear samples and controls.

When a larger number of reference genes is used, the SD of the normalization factor (mean of reference gene expression) is reduced, and the random variation among the expression of the tested genes is partially cancelled. In using the GenEx software, we observed that in most of the analysis groups, the Acc.SD value of 2 reference genes differed by no more than 0.1 from that observed when 3 or more reference genes were used. Because the inclusion of additional reference genes increases the time and money required for the analysis, it is important to consider the degree of improvement and overall noise contributed by reference genes when deciding how many reference genes are required. The design of the study always need to be consider; however, taking together all the results shown in this study, we suggest that 3 or more reference genes should be used for gene expression normalization in ACL samples.

It is important to note that *ACTB + HPRT1 + 18S* was the best trio for the analysis involving isolated ACL tear samples and controls. On the other hand, *ACTB + TBP+ 18S* was the best trio in the analysis involving ACL tear samples of patients with a concomitant meniscal tear and controls. Therefore, if the gene expression study aims to compare non-injured ACL, isolated ACL tears and ACL tears from patients with meniscal tear as three independent groups, four reference genes should be used. *ACTB + TBP + HPRT1 + 18S* were the top ranked stable genes in most of the analysis (S1 Table).

Additionally, we evaluated the effect of the use of different combinations of reference genes in the expression of 9 other extracellular matrix genes (data not shown). The analysis reinforces that it is not appropriate to use only one reference gene for gene expression normalization in the study of ACL samples. Furthermore, for the studied genes, no significant difference was found between isolated ACL tear samples and the ACL samples of patients with meniscal tears.

Our study presented some limitations. First, we included only a limited number of candidate reference genes; it is likely that some other genes may also be used as internal references for gene expression studies in ACL samples from patients with or without a history of ACL tear. Second, the number of samples available for the independent t-test was reduced, especially in the control group. However, to the best of our knowledge, only one previous study evaluated the RNA expression in human non-injured ACL samples [9]. Third, our results apply directly only to ACL samples. It is unclear how well our results could be extended to other joint ligaments. Therefore, when other ligament samples are used, we suggest doing specific gene expression studies to identify the most stable reference genes for normalization. Nevertheless, it is important to highlight that our results may be relevant to the study of both ACL tears and normal ACL.

## Conclusions

The results of the present study indicate that the use of suitable reference genes for reliable gene expression evaluation by RT-qPCR should consider the type of ACL samples investigated (injured or non-injured). Based on the evaluation of different analysis groups, *ACTB* seems to be the most suitable reference gene and *ACTB + TBP* seems to be the best pair of reference genes. However, the use of only one or two reference genes does not seem suitable for gene expression normalization in ACL tear studies. *ACTB+HPRT1+18S* is the best trio for the analyses involving isolated ACL tears and controls. Conversely, *ACTB+TBP+18S* is the best trio for the analyses involving (1) injured ACL tears and controls, and (2) ACL tears of patients with meniscal tears and controls. Therefore, if the gene expression study aims to compare non-injured ACL, isolated ACL tears and ACL tears from patients with meniscal tear as three independent groups*ACTB+TBP+18S+HPRT1* should be used. The results of this work may benefit future studies on ACL that require more accurate gene expression quantification.

## Supporting Information

S1 Table Ranking of the candidate single reference genes by each method used. (DOCX)

## References

[1] Arliani GG, Astur DC, Moraes ER, Kaleka CC, Jalikjian W, et al (2012) Three dimensional anatomy of the anterior cruciate ligament: a new approach in anatomical orthopedic studies and a literature review. Open Access J Sports Med 3: 183–188. 10.2147/OAJSM.S37203 24198601 PMC3781913

[2] Arliani GG, Astur DC, Kanas M, Kaleka CC, Cohen M (2012) Anterior cruciate ligament injury: treatment and rehabilitation. Current perspectives and trends. Rev Bras Ortop 47: 6. 10.1016/S2255-4971(15)30085-9 PMC4799385 27042620

[3] Astur DC, Santos CV, Aleluia V, Astur Neto N, Arliani GG, et al (2013) Characterization of cruciate ligament impingement: the influence of femoral or tibial tunnel positioning at different degrees of knee flexion. Arthroscopy 29: 913–919. 10.1016/j.arthro.2013.01.008 23419357

[4] Astur DC, Aleluia V, Santos CV, Arliani GG, Badra R, et al (2012) Risks and consequences of using the transportal technique in reconstructing the anterior cruciate ligament: relationships between the femoral tunnel, lateral superior genicular artery and lateral epicondyle of the femoral condyle. Rev Bras Ortop 47: 5. 10.1016/S2255-4971(15)30011-2 PMC4799463 27047873

[5] Astur DC, Aleluia V, Veronese C, Astur N, Oliveira SG, et al (2014) A prospective double blinded randomized study of anterior cruciate ligament reconstruction with hamstrings tendon and spinal anesthesia with or without femoral nerve block. Knee 21: 911–915. 10.1016/j.knee.2014.06.003 24993276

[6] da Silveira Franciozi CE, Ingham SJ, Gracitelli GC, Luzo MV, Fu FH, et al (2014) Updates in biological therapies for knee injuries: anterior cruciate ligament. Curr Rev Musculoskelet Med 7: 228–238. 10.1007/s12178-014-9228-9 25070265 PMC4596162

[7] Astur DC, Lauxen D, Ejnisman B, Cohen M (2014) Twin athlete brothers with open physes operated for ACL reconstruction on the same day, but with different elapsed times after injury: a 5-year follow-up. BMJ Case Rep 2014. 10.1136/bcr-2013-202650 PMC3918611 24510695

[8] Johnson JS, Morscher MA, Jones KC, Moen SM, Klonk CJ, et al (2015) Gene expression differences between ruptured anterior cruciate ligaments in young male and female subjects. J Bone Joint Surg Am 97: 71–79. 10.2106/JBJS.N.00246 25568397

[9] Lo IK, Marchuk LL, Hart DA, Frank CB (1998) Comparison of mRNA levels for matrix molecules in normal and disrupted human anterior cruciate ligaments using reverse transcription-polymerase chain reaction. J Orthop Res 16: 421–428. 9747782 10.1002/jor.1100160405

[10] Naraoka T, Ishibashi Y, Tsuda E, Yamamoto Y, Kusumi T, et al (2012) Time-dependent gene expression and immunohistochemical analysis of the injured anterior cruciate ligament. Bone Joint Res 1: 238–244. 10.1302/2046-3758.110.2000118 23610654 PMC3626253

[11] Spindler KP, Clark SW, Nanney LB, Davidson JM (1996) Expression of collagen and matrix metalloproteinases in ruptured human anterior cruciate ligament: an in situ hybridization study. J Orthop Res 14: 857–861. 8982126 10.1002/jor.1100140603

[12] Lo IK, Marchuk L, Hart DA, Frank CB (2003) Messenger ribonucleic acid levels in disrupted human anterior cruciate ligaments. Clin Orthop Relat Res: 249–258. 12567153 10.1097/00003086-200302000-00034

[13] Derveaux S, Vandesompele J, Hellemans J (2010) How to do successful gene expression analysis using real-time PCR. Methods 50: 227–230. 10.1016/j.ymeth.2009.11.001 19969088

[14] Kozera B, Rapacz M (2013) Reference genes in real-time PCR. J Appl Genet 54: 391–406. 24078518 10.1007/s13353-013-0173-x PMC3825189

[15] Li YL, Ye F, Hu Y, Lu WG, Xie X (2009) Identification of suitable reference genes for gene expression studies of human serous ovarian cancer by real-time polymerase chain reaction. Anal Biochem 394: 110–116. 10.1016/j.ab.2009.07.022 19622337

[16] Yuzbasioglu A, Onbasilar I, Kocaefe C, Ozguc M (2010) Assessment of housekeeping genes for use in normalization of real time PCR in skeletal muscle with chronic degenerative changes. Exp Mol Pathol 88: 326–329. 10.1016/j.yexmp.2009.12.007 20045408

[17] Leal MF, Belangero PS, Cohen C, Figueiredo EA, Loyola LC, et al (2014) Identification of suitable reference genes for gene expression studies of shoulder instability. PLoS One 9: e105002 10.1371/journal.pone.0105002 25122470 PMC4133370

[18] Leal MF, Belangero PS, Figueiredo EA, Cohen C, Loyola LC, et al (2015) Identification of suitable reference genes for gene expression studies in tendons from patients with rotator cuff tear. PLoS One 10: e0118821 10.1371/journal.pone.0118821 25768100 PMC4358921

[19] Pombo-Suarez M, Calaza M, Gomez-Reino JJ, Gonzalez A (2008) Reference genes for normalization of gene expression studies in human osteoarthritic articular cartilage. BMC Mol Biol 9: 17 10.1186/1471-2199-9-17 18226276 PMC2248200

[20] Zhou ZJ, Zhang JF, Xia P, Wang JY, Chen S, et al (2014) Selection of suitable reference genes for normalization of quantitative real-time polymerase chain reaction in human cartilage endplate of the lumbar spine. PLoS One 9: e88892 10.1371/journal.pone.0088892 24558443 PMC3928306

[21] Ayers D, Clements DN, Salway F, Day PJ (2007) Expression stability of commonly used reference genes in canine articular connective tissues. BMC Vet Res 3: 7 17484782 10.1186/1746-6148-3-7 PMC1884148

[22] Torg JS, Conrad W, Kalen V (1976) Clinical diagnosis of anterior cruciate ligament instability in the athlete. Am J Sports Med 4: 84–93. 961972 10.1177/036354657600400206

[23] Marshall JL, Wang JB, Furman W, Girgis FG, Warren R (1975) The anterior drawer sign: what is it? J Sports Med 3: 152–158. 1219194 10.1177/036354657500300402

[24] Galway R, Beaupre A, McIntosh DL (1972) Pivot Shift—a clinical sign of symptomatic anterior cruciate insufficiency. J Bone Joint Surg (Br) 54: 2.

[25] McMurray TP (1949) The semilunar cartilages. Br J Surg 29: 1.

[26] Apley AG (1947) The diagnosis of meniscal injuries: Some new clinical methods. J Bone Joint Surg 29: 7. 20284687

[27] Tria AJ, Klein KS (1992) An illustrated guide to the knee. New York: Churchill Livingstone.

[28] Speziali A, Placella G, Tei MM, Georgoulis A, Cerulli G (2015) Diagnostic value of the clinical investigation in acute meniscal tears combined with anterior cruciate ligament injury using arthroscopic findings as golden standard. Musculoskelet Surg. 10.1007/s12306-015-0348-1 25683263

[29] Taylor SC, Mrkusich EM (2014) The state of RT-quantitative PCR: firsthand observations of implementation of minimum information for the publication of quantitative real-time PCR experiments (MIQE). J Mol Microbiol Biotechnol 24: 46–52. 10.1159/000356189 24296827

[30] Vakonakis I, Campbell ID (2007) Extracellular matrix: from atomic resolution to ultrastructure. Curr Opin Cell Biol 19: 578–583. 17942296 10.1016/j.ceb.2007.09.005 PMC4827755

[31] Eyre DR, Koob TJ, Van Ness KP (1984) Quantitation of hydroxypyridinium crosslinks in collagen by high-performance liquid chromatography. Anal Biochem 137: 380–388. 6731820 10.1016/0003-2697(84)90101-5

[32] Myllyla R, Wang C, Heikkinen J, Juffer A, Lampela O, et al (2007) Expanding the lysyl hydroxylase toolbox: new insights into the localization and activities of lysyl hydroxylase 3 (LH3). J Cell Physiol 212: 323–329. 17516569 10.1002/jcp.21036

[33] Silver N, Best S, Jiang J, Thein SL (2006) Selection of housekeeping genes for gene expression studies in human reticulocytes using real-time PCR. BMC Mol Biol 7: 33 17026756 10.1186/1471-2199-7-33 PMC1609175

[34] Andersen CL, Jensen JL, Orntoft TF (2004) Normalization of real-time quantitative reverse transcription-PCR data: a model-based variance estimation approach to identify genes suited for normalization, applied to bladder and colon cancer data sets. Cancer Res 64: 5245–5250. 15289330 10.1158/0008-5472.CAN-04-0496

[35] Vandesompele J, De Preter K, Pattyn F, Poppe B, Van Roy N, et al (2002) Accurate normalization of real-time quantitative RT-PCR data by geometric averaging of multiple internal control genes. Genome Biol 3: RESEARCH0034 12184808 10.1186/gb-2002-3-7-research0034 PMC126239

[36] Pfaffl MW, Tichopad A, Prgomet C, Neuvians TP (2004) Determination of stable housekeeping genes, differentially regulated target genes and sample integrity: BestKeeper—Excel-based tool using pair-wise correlations. Biotechnol Lett 26: 509–515. 15127793 10.1023/b:bile.0000019559.84305.47

[37] Huggett J, Dheda K, Bustin S, Zumla A (2005) Real-time RT-PCR normalisation; strategies and considerations. Genes Immun 6: 279–284. 15815687 10.1038/sj.gene.6364190

[38] Lyng MB, Laenkholm AV, Pallisgaard N, Ditzel HJ (2008) Identification of genes for normalization of real-time RT-PCR data in breast carcinomas. BMC Cancer 8: 20 10.1186/1471-2407-8-20 18211679 PMC2248196

[39] Rubie C, Kempf K, Hans J, Su T, Tilton B, et al (2005) Housekeeping gene variability in normal and cancerous colorectal, pancreatic, esophageal, gastric and hepatic tissues. Mol Cell Probes 19: 101–109. 15680211 10.1016/j.mcp.2004.10.001

[40] Fu J, Bian L, Zhao L, Dong Z, Gao X, et al (2010) Identification of genes for normalization of quantitative real-time PCR data in ovarian tissues. Acta Biochim Biophys Sin (Shanghai) 42: 568–574. 20705598 10.1093/abbs/gmq062

[41] Wang Q, Ishikawa T, Michiue T, Zhu BL, Guan DW, et al (2012) Stability of endogenous reference genes in postmortem human brains for normalization of quantitative real-time PCR data: comprehensive evaluation using geNorm, NormFinder, and BestKeeper. Int J Legal Med 126: 943–952. 10.1007/s00414-012-0774-7 23010907

[42] Wisnieski F, Calcagno DQ, Leal MF, dos Santos LC, Gigek Cde O, et al (2013) Reference genes for quantitative RT-PCR data in gastric tissues and cell lines. World J Gastroenterol 19: 7121–7128. 10.3748/wjg.v19.i41.7121 24222956 PMC3819548

[43] Llanos A, François JM, Parrou J-L Tracking the best reference genes for RT-qPCR data normalization in filamentous fungi. BMC Genomics 16. 10.1186/s12864-015-1224-y PMC4342825 25757610

[44] de Jonge HJ, Fehrmann RS, de Bont ES, Hofstra RM, Gerbens F, et al (2007) Evidence based selection of housekeeping genes. PLoS One 2: e898 17878933 10.1371/journal.pone.0000898 PMC1976390

[45] Killian ML, Isaac DI, Haut RC, Dejardin LM, Leetun D, et al (2010) Traumatic anterior cruciate ligament tear and its implications on meniscal degradation: a preliminary novel lapine osteoarthritis model. J Surg Res 164: 234–241. 10.1016/j.jss.2009.03.006 19577765

