# Supplementary material for: Identification of Suitable Reference Genes for Investigating Gene Expression in Anterior Cruciate Ligament Injury by Using Reverse Transcription-Quantitative PCR
Source: PLoS One. 2015 Jul 20;10(7):e0133323. doi: 10.1371/journal.pone.0133323 (PMC4507999; doi:10.1371/journal.pone.0133323)
Supplement: S1 Table — (DOCX) [file pone.0133323.s001.docx]

**S1 Table. Ranking of the candidate single reference genes by each method used.**

| **NormFinder** | |  | **geNorm** | |  | **BestKeeper** | |  | **DataAssist** | |  | **ΔCt method** | |  | **RefFinder** | |
| --- | --- | --- | --- | --- | --- | --- | --- | --- | --- | --- | --- | --- | --- | --- | --- | --- |
| **Stability value*** | **Ranking** |  | **M value*** | **Ranking** |  | **CV*** | **Ranking** |  | **Score*** | **Ranking** |  | **Mean SD*** | **Ranking** |  | **Geomean*** | **Ranking** |
| Isolated ACL tear samples | | | | | | | | | | | | | | | | |
| 0.1909 | *HPRT1* |  | 0.2974 | *ACTB* |  | 2.79 | *TBP* |  | 0.3973 | *ACTB* |  | 0.52 | *HPRT1* |  | 1.68 | *HPRT1* |
| 0.1936 | *ACTB* |  | 0.2974 | *18S* |  | 2.87 | *HPRT1* |  | 0.4060 | *HPRT1* |  | 0.56 | *ACTB* |  | 2.21 | *18S* |
| 0.2610 | *18S* |  | 0.3552 | *TBP* |  | 4.09 | *B2M* |  | 0.4209 | *18S* |  | 0.56 | *TBP* |  | 2.28 | *TBP* |
| 0.3120 | *TBP* |  | 0.3724 | *B2M* |  | 4.22 | *ACTB* |  | 0.4616 | *B2M* |  | 0.56 | *18S* |  | 2.38 | *ACTB* |
| 0.3531 | *B2M* |  | 0.3787 | *HPRT1* |  | 4.52 | *GAPDH* |  | 0.4457 | *TBP* |  | 0.64 | *GAPDH* |  | 5.23 | *GAPDH* |
| 0.5380 | *GAPDH* |  | 0.4449 | *GAPDH* |  | 8.01 | *18S* |  | 0.5937 | *GAPDH* |  | 0.66 | *B2M* |  | 5.73 | *B2M* |
| ACL tear samples of patients with a concomitant meniscal tear | | | | | | | | | | | | | | | | |
| 0.1091 | *ACTB* |  | 0.4347 | *ACTB* |  | 3.72 | *HPRT1* |  | 0.5662 | *ACTB* |  | 0.67 | *ACTB* |  | 1 | *ACTB* |
| 0.2720 | *TBP* |  | 0.4347 | *TBP* |  | 4.07 | *TBP* |  | 0.5962 | *TBP* |  | 0.73 | *TBP* |  | 1.86 | *TBP* |
| 0.4902 | *18S* |  | 0.5315 | *18S* |  | 5.57 | *GAPDH* |  | 0.6994 | *18S* |  | 0.81 | *18S* |  | 3.66 | *18S* |
| 0.0612 | *GAPDH* |  | 0.5871 | *GAPDH* |  | 6.38 | *ACTB* |  | 0.7560 | *GAPDH* |  | 0.87 | *GAPDH* |  | 4.23 | *GAPDH* |
| 0.6385 | *HPRT1* |  | 0.6412 | *HPRT1* |  | 6.76 | *B2M* |  | 0.7900 | *B2M* |  | 0.90 | *B2M* |  | 4.56 | *HPRT1* |
| 0.6628 | *B2M* |  | 0.6877 | *B2M* |  | 12.31 | *18S* |  | 0.8258 | *HPRT1* |  | 0.93 | *HPRT1* |  | 4.61 | *B2M* |
| ACL controls | | | | | | | | | | | | | | | | |
| 0.2834 | *ACTB* |  | 0.3022 | *HPRT1* |  | 2.09 | *TBP* |  | 0.4659 | *ACTB* |  | 0.55 | *ACTB* |  | 1.57 | *ACTB* |
| 0.3143 | *18S* |  | 0.3022 | *GAPDH* |  | 2.24 | *HPRT1* |  | 0.4769 | *HPRT1* |  | 0.57 | *HPRT1* |  | 1.86 | *TBP* |
| 0.3360 | *HPRT1* |  | 0.4074 | *18S* |  | 2.70 | *GAPDH* |  | 0.4823 | *18S* |  | 0.63 | *TBP* |  | 2.63 | *HPRT1* |
| 0.3441 | *GAPDH* |  | 0.4304 | *ACTB* |  | 3.61 | *ACTB* |  | 0.4992 | *GAPDH* |  | 0.63 | *B2M* |  | 3.94 | *B2M* |
| 0.4294 | *B2M* |  | 0.4779 | *B2M* |  | 4.16 | *B2M* |  | 0.5404 | *TBP* |  | 0.63 | *18S* |  | 4.40 | *18S* |
| 0.4300 | *TBP* |  | 0.5020 | *TBP* |  | 6.57 | *18S* |  | 0.5622 | *B2M* |  | 0.67 | *GAPDH* |  | 4.56 | *GAPDH* |
| All injured ACL samples | | | | | | | | | | | | | | | | |
| 0.1521 | *ACTB* |  | 0.4147 | *ACTB* |  | 3.33 | *HPRT1* |  | 0.4813 | *ACTB* |  | 0.62 | *ACTB* |  | 1.00 | *ACTB* |
| 0.3140 | *TBP* |  | 0.4147 | *TBP* |  | 3.40 | *TBP* |  | 0.5371 | *TBP* |  | 0.66 | *TBP* |  | 1.86 | *TBP* |
| 0.4007 | *18S* |  | 0.4671 | *18S* |  | 5.09 | *GAPDH* |  | 0.5681 | *18S* |  | 0.71 | *18S* |  | 3.13 | *HPRT1* |
| 0.4547 | *HPRT1* |  | 0.5058 | *B2M* |  | 5.35 | *B2M* |  | 0.6639 | *GAPDH* |  | 0.74 | *HPRT1* |  | 3.66 | *18S* |
| 0.5313 | *B2M* |  | 0.5439 | *HPRT1* |  | 5.38 | *ACTB* |  | 0.6430 | *B2M* |  | 0.77 | *GAPDH* |  | 4.73 | *GAPDH* |
| 0.5680 | *GAPDH* |  | 0.5866 | *GAPDH* |  | 10.21 | *18S* |  | 0.6294 | *HPRT1* |  | 0.79 | *B2M* |  | 6.00 | *B2M* |
| Isolated ACL tear samples and controls | | | | | | | | | | | | | | | | |
| 0.2475 | *ACTB* |  | 0.3537 | *ACTB* |  | 2.59 | *TBP* |  | 0.4409 | *ACTB* |  | 0.57 | *ACTB* |  | 1.41 | *ACTB* |
| 0.2758 | *18S* |  | 0.3537 | *18S* |  | 2.62 | *HPRT1* |  | 0.4539 | *18S* |  | 0.57 | *HPRT1* |  | 2.28 | *18S* |
| 0.2914 | *HPRT1* |  | 0.4174 | *TBP* |  | 3.80 | *GAPDH* |  | 0.4609 | *HPRT1* |  | 0.59 | *18S* |  | 2.38 | *HPRT1* |
| 0.3581 | *TBP* |  | 0.4285 | *B2M* |  | 3.91 | *ACTB* |  | 0.4942 | *TBP* |  | 0.61 | *TBP* |  | 2.63 | *TBP* |
| 0.3986 | *B2M* |  | 0.4489 | *HPRT1* |  | 4.22 | *B2M* |  | 0.5201 | *B2M* |  | 0.65 | *B2M* |  | 5.23 | *B2M* |
| 0.4770 | *GAPDH* |  | 0.4851 | *GAPDH* |  | 7.42 | *18S* |  | 0.5731 | *GAPDH* |  | 0.66 | *GAPDH* |  | 5.73 | *GAPDH* |
| ACL tear samples of patients with a concomitant meniscal tear and controls | | | | | | | | | | | | | | | | |
| 0.2030 | *ACTB* |  | 0.4529 | *ACTB* |  | 3.12 | *HPRT1* |  | 0.5346 | *ACTB* |  | 0.62 | *ACTB* |  | 1.19 | *ACTB* |
| 0.3414 | *TBP* |  | 0.4529 | *18S* |  | 3.29 | *TBP* |  | 0.577 | *TBP* |  | 0.69 | *TBP* |  | 2.00 | *TBP* |
| 0.4319 | *18S* |  | 0.5212 | *TBP* |  | 4.43 | *GAPDH* |  | 0.6286 | *18S* |  | 0.75 | *18S* |  | 2.99 | *GAPDH* |
| 0.5146 | *GAPDH* |  | 0.5581 | *GAPDH* |  | 5.31 | *ACTB* |  | 0.674 | *GAPDH* |  | 0.80 | *GAPDH* |  | 3.66 | *18S* |
| 0.5482 | *HPRT1* |  | 0.5932 | *HPRT1* |  | 5.74 | *B2M* |  | 0.7126 | *B2M* |  | 0.80 | *B2M* |  | 4.61 | *B2M* |
| 0.5871 | *B2M* |  | 0.6300 | *B2M* |  | 10.09 | *18S* |  | 0.7181 | *HPRT1* |  | 0.81 | *HPRT1* |  | 5.05 | *HPRT1* |
| All ACL samples | | | | | | | | | | | | | | | | |
| 0.2041 | *ACTB* |  | 0.4162 | *ACTB* |  | 3.06 | *HPRT1* |  | 0.4913 | *ACTB* |  | 0.61 | *ACTB* |  | 1.00 | *ACTB* |
| 0.3401 | *TBP* |  | 0.4162 | *18S* |  | 3.11 | *TBP* |  | 0.5405 | *TBP* |  | 0.66 | *TBP* |  | 1.86 | *TBP* |
| 0.3824 | *18S* |  | 0.4756 | *TBP* |  | 4.49 | *GAPDH* |  | 0.5673 | *18S* |  | 0.69 | *18S* |  | 3.13 | *HPRT1* |
| 0.4463 | *HPRT1* |  | 0.5003 | *B2M* |  | 4.89 | *ACTB* |  | 0.6205 | *HPRT1* |  | 0.72 | *HPRT1* |  | 3.66 | *18S* |
| 0.5072 | *B2M* |  | 0.5419 | *HPRT1* |  | 5.10 | *B2M* |  | 0.6313 | *B2M* |  | 0.74 | *GAPDH* |  | 4.73 | *GAPDH* |
| 0.5201 | *GAPDH* |  | 0.5734 | *GAPDH* |  | 9.27 | *18S* |  | 0.6466 | *GAPDH* |  | 0.75 | *B2M* |  | 6.00 | *B2M* |

*A lower value indicates higher stability in gene expression. ACL: anterior cruciate ligament.
